# Peptide Extracts from Seven Medicinal Plants Discovered to Inhibit Oomycete *Phytophthora infestans,* a Causative Agent of Potato Late Blight Disease

**DOI:** 10.3390/plants9101294

**Published:** 2020-09-30

**Authors:** Eugene A. Rogozhin, Alexey S. Vasilchenko, Anna S. Barashkova, Alexey N. Smirnov, Sergey K. Zavriev, Vladimir P. Demushkin

**Affiliations:** 1 Shemyakin and Ovchinnikov Institite of Bioorganic Chemistry Russian Academy of Sciences, 117997 Moscow, Russia; barashkova.an@gmail.com (A.S.B.); szavriev@ibch.ru (S.K.Z.); vpdem@ibch.ru (V.P.D.); 2 Gause Institute of New Antibiotics, 119021 Moscow, Russia; 3 All-Russian Institute of Plant Protection, 196608 St.-Petersburg-Pushkin, Russia; 4 Institute of Biological and Agricultural Biology (X-Bio) Tyumen State University, Russian Federation, 625003 Tyumen, Russia; a.s.vasilchenko@utmn.ru; 5 Timiryazev Russian State Agrarian University, 127550 Moscow, Russia; smirnov@timacad.ru

**Keywords:** medicinal plants, amino acid composition, liquid chromatography, anti-oomycete activity, *Phytophthora infestans*, peptide fragments, protein hydrolysis

## Abstract

We report the inhibitory effect of peptide extracts obtained from seven medicinal plants against a causative agent of late blight disease *Phytophthora infestans.* We find that all the extracts possess inhibitory activity toward the zoospores output, zoosporangium germination, and the development of *P. infestans* on potato disc tubers at different quantitative levels. Based on the biological effects detected, an extract of common horsetail (*Equisetum arvense*) biomass is recognized as the most effective and is selected for further structural analysis. We perform a combination of amino acid analysis and MALDI-TOF mass spectrometry, which reveal the presence of Asn/Asp- and Gln/Glu-rich short peptides with molecular masses in the range of 500–900 Da and not exceeding 1500 Da as the maximum. Analytical anion-exchange HPLC is successfully applied for separation of the peptide extract from common horsetail (*E. arvense*). We collect nine dominant components that are combined in two groups with differences in retention times. The *N*-terminal amino acid sequence of the prevalent compounds after analytical ion-exchange HPLC allows us to identify them as peptide fragments of functionally active proteins associated with photosynthesis, aquatic transport, and chitin binding. The anti-oomycete effects may be associated with the conversion of ribulose-1,5-bisphosphate carboxylase/oxygenase to produce a number of biologically active anionic peptides with possible regulatory functions. These data inform our knowledge regarding biologically active peptide fragments; they are the components of programmed or induced proteolysis of plant proteins and can realize secondary antimicrobial functions.

## 1. Introduction

Plant diseases caused by oomycetes are a primary problem for agriculture in the world [1]. Late blight disease is one of the most important and dangerous potato and tomato diseases with the potential for practical annihilation of potato plants [2,3]. The pathogen affects plants in damp conditions and causes necrosis of the leaves and rapid tuber decomposition [3,4]. Late blight can progress very quickly in both organic and traditional agriculture, even with extensive fungicide application. Phytopathogenic fungi and oomycetes can be killed via alternative agents [5,6,7,8] with limited environmental pollution, unlike traditional pesticides. This is important as pathogenic microorganisms have become increasingly resistant to various chemical pesticides.

Renewable resources are generally preferred based on sustainability [9,10,11]. Many studies have evaluated natural variants regarding the limitation of late blight including antagonistic systems based on the application of other fungal species [12] and the participation of rhizosphere bacteria in the defense system of tomatoes from late blight disease [13,14,15,16,17]. Other approaches are based on the application of micro- and nano-particles from natural polymers to control the oomycete development from the *Phytophthora* genus [18,19]. There are currently many bioactive substances against phytopathogenic organisms, but they do not typically have a stable effect against late blight inhibition [20,21].

Other options include composts [20,22], fungal secondary metabolites [23,24,25,26], and plant derivation [27]. Coniferous and flowering plant ethanol extracts [28,29], including lichens [30], have been used also. Crude plant extracts also can have antifungal properties [31,32,33]. Thus, scientific research on the plant extracts is required to better understand their efficacy and the mechanism to control diseases and insect pests of cultivated plants in the majority of developing countries [34]. The antitumor activity of peptide extracts from medicinal plants was previously shown [35,36]. Here, we screen plant peptide extracts that suppress agents of late blight and perform purification and biochemical analysis on the most active variant.

## 2. Results

### 2.1. Screening of Plant Peptide Extracts to Suppress Phytophthora infestans In Vitro

**Figure 1:**
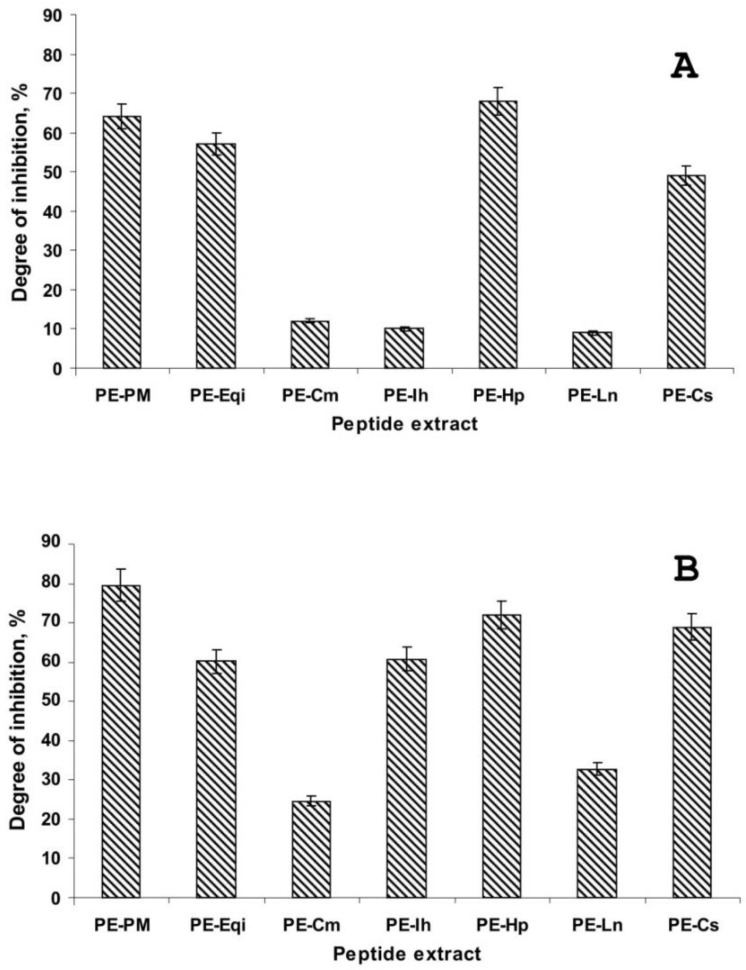
The antimicrobial activity of the plant peptide extracts against *Phytophthora infestans* by optical microscopy: (**A**)—indirect germination, (**B**)—direct germination.

During this study, a number of medicinal plants that are donors of peptide extracts were selected for the following screening against the late blight disease oomycete *Phytophthora infestans*. Here, we successfully developed a method with limited acid hydrolysis of the total protein content to obtain a standardized composition revealing the biological activity independently. To explore the antimicrobial activity of the peptide extracts, we applied their influence on the indirect and direct germination of *P. infestans* zoosporangia in laboratory conditions using optical microscopy. We performed a two-fold dilution series for experimental measurements of the IC_50_ and IC_min_ values (Table 1). The most pronounced inhibition was evaluated using the direct germination of zoosporangia using a growing tube at an IC_min_ from 0.25 to 2.0 mg/mL. The mixture of three plants (PE-PM) was two-fold more active (0.125 mg/mL) than the extracts obtained from the individual plants (PE-Hp and PE-Cs). This displayed an equal activity against indirect germination (output of zoospores from the zoosporangia) compared with the other variants tested to reveal essentially less biological action. The antimicrobial effects of all peptide extracts were lower relative to the direct and indirect oomycete germination (0.5–2.0 mg/mL) based on the IC_50_ values; however, the peptide extract in a mixture demonstrated significantly reduced IC_50_ values (0.25 mg/mL) (Figure 1).

Additionally, some morphological changes of the oomycete structures were observed after incubation with the peptide extracts. The PE-PM at a maximal concentration (2 mg/mL) caused partial destruction and covering lysis of 18% fungal cells in the treated sample at a level of 15–18% (Table 1). However, there was often no deformation of the zoosporangia, zoospores, and mycelium when a single plant extract was added.

### 2.2. The Antifungal Activity of Plant Peptide Extracts toward P. infestans by Inoculation of Potato Tuber Discs

**Figure 2:**
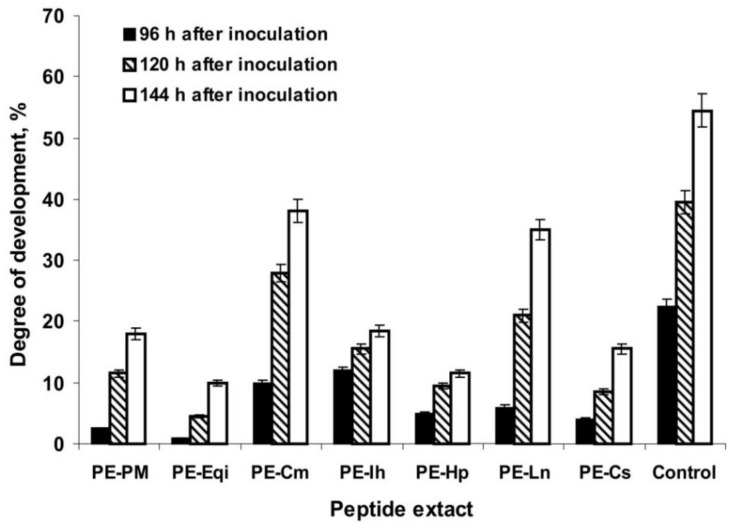
Antimicrobial activity of the plant peptide extracts against *P. infestans* by inoculation of potato tuber slices.

Peptide extracts inhibited *P. infestans* in vitro at a different level. We evaluated the growth and development of the oomycete when it was inoculated on the surface of potato tuber discs. This method was more objective for understanding the real three-sided interaction between active peptides inside the extracts and understanding the phytopathogen and plant tissue responsible for inoculation from plant innate immunity. The results showed the ability of the peptide extracts to inhibit the appearance of *P. infestans* symptoms (necrotic spots and sporiferous layers) even 96 h after inoculation: thus, the average area of the potato disc damage was measured to be less than 5% (excepting PE-Cm and PE-Ih) relative to the control (approximately 20% of the oomycete development) at the highest concentration tested (2 mg/mL) (Figure 2). PE-Eqi could induce nearly the complete inhibition of *P. infestans* symptoms. After 120 h of incubation, the peptide extracts retained a degree of inhibition relative to the positive control (approximately 40% of the oomycete development on the potato disc surface).

**Figure 3:**
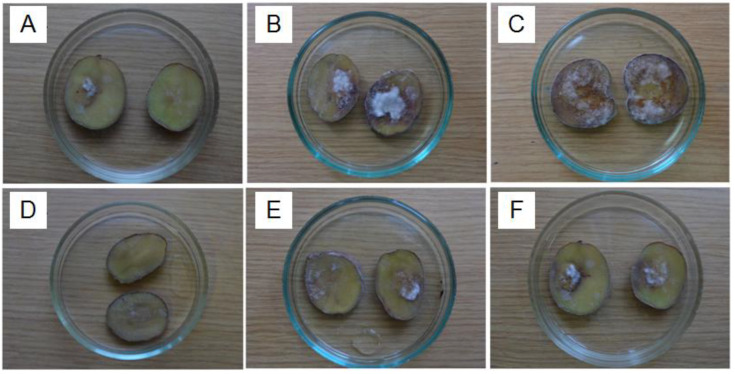
Dynamics of late blight disease development on potato tuber discs: (**A**)—control since 96 h after inoculation, (**B**)—control since 120 h after inoculation, (**C**)—control since 144 h after inoculation; (**D**)—PE-Eqi since 96 h after inoculation, (**E**)—PE-Eqi since 120 h after inoculation, (**F**)—PE-Eqi since 144 h after inoculation.

Finally, most variants effectively suppressed *P. infestans* on the plant tissue within 144 h of incubation (a final registration point). The peptide extract from common horsetail (*E. arvense)* revealed the most pronounced effect in a time-dependent manner (Figure 3). Thus, combining the results of two independent assays suggested that the peptide extracts from certain medicinal plants can act toward *P. infestans* at a different level. This is likely associated with the composition of polypeptides that is unique to each plant. 

### 2.3. Initial Structural Characterization of PE-Eqi

**Figure 4:**
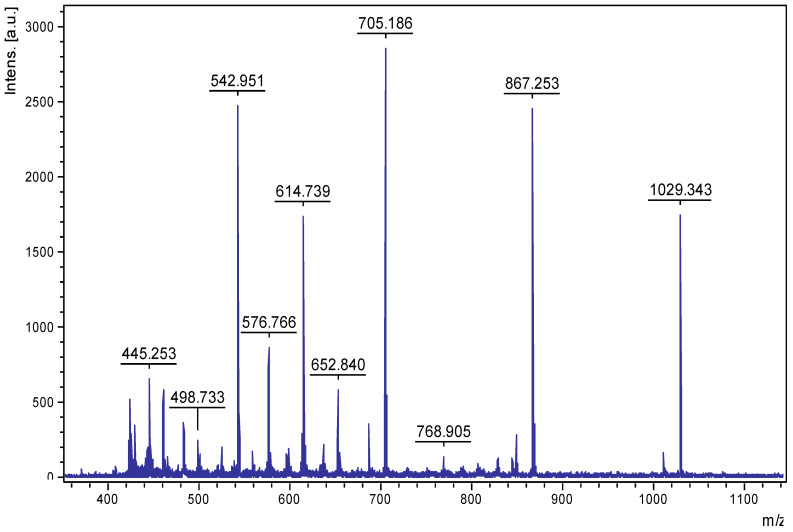
MALDI-TOF MS analysis of the extract from common horsetail (*E. arvense*). Intens. [a.u.]—intensity of *m*/*z* signal, absorption units.

Based on the screening results, PE-Eqi from the common horsetail (*E. arvense*) was selected for further investigation. PE-Eqi had the most pronounced inhibition activity of all individual plants and contributed to a high level of activity in the PE-Pm mixture. PE-Eqi was acknowledged to be the most attractive concentrate; it contained a set of polypeptides with the highest activity to suppress *P. infestans*. Thus, it was a resultant candidate to be an active substance for a novel biopesticide. The total yield of polypeptides in this method was 110–120 mg per 100 g of plant material. The amino acid composition of PE-Eqi led to a significant content of Gln/Glu and Asn/Asp (more than 40% from the total weight); these were presented as dicarboxylic acids. The total peptide material contained about 90% of the extract mass. MALDI-MS analysis of the total peptide showed that the most intensive *m*/*z* signals were measured in the range of 500–900 Da and did not exceed 1500 Da as the maximum (Figure 4). This led to a specified type of limited acidic hydrolysis to obtain reasonably short polypeptides.

### 2.4. HPLC Analysis of PE-Eqi and Structure Determination of Individual Compounds

**Figure 5:**
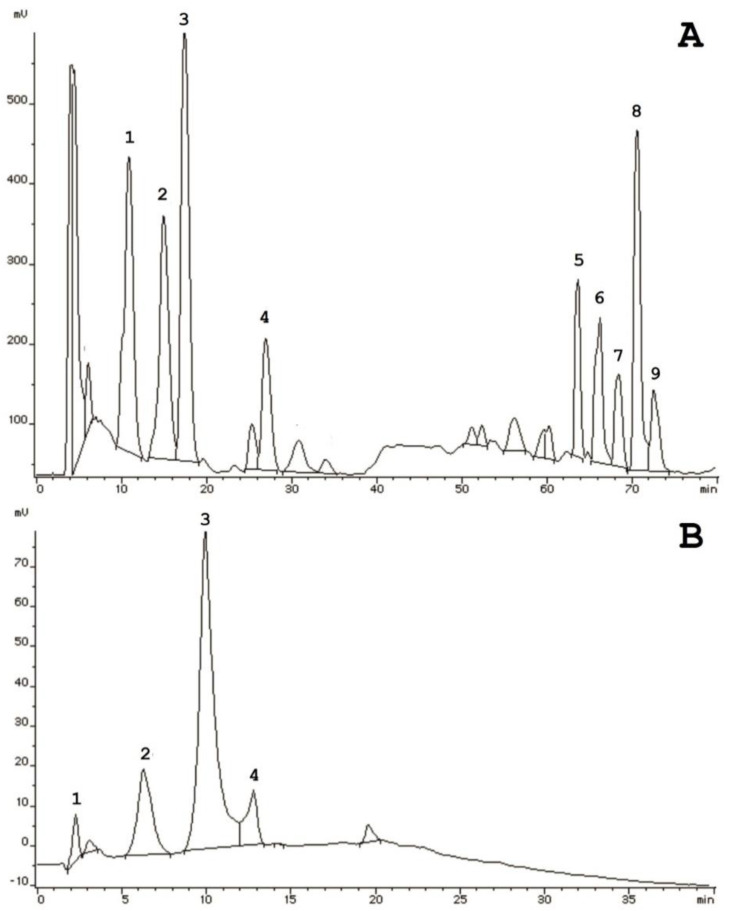
Chromatographic analysis of the PE-Eqi extract: (**A**)—separation by anion-exchange HPLC, (**B**)—separation by reversed-phase HPLC.

The next stage of investigation was to separate the PE-Eqi via analytical HPLC. The peptide extract was initially applied on a reverse-phase column for further fractionation over a linear gradient of organic solvents; however, we did not show any qualitative separation in resolution. Thus, there were only four main peaks visualized in the profile (Figure 5B). Subsequentially, we performed ion-exchange HPLC and attempted to disperse compounds that were localized in the extract using the anion-exchange immobile phase. The amino acid analysis suggested a prevalence of dicarboxylic acids that were negatively charged. Therefore, we could discover individual components combined into the two main groups: high hydrophilic and high hydrophobic (Figure 5A). Thus, nine compounds were manually collected in which four (№ 1–4) were eluted from the column from 10 to 30 min, and five of them (№ 5–9) had a pronounced retention time (65–75 min).

Our approach could achieve high-resolution separation of the compounds that differed via the presence of negatively charged groups (hypothetically hydroxyls) unlike the separation of hydrophobic liquid chromatography. To provide structural identification of the peptide components located inside the PE-Eqi extract, all samples were manually collected and analyzed with MALDI-TOF MS; N-terminal amino acid sequencing using the Edman automated degradation technique. The sequences were selected for homology searching in NCBI databases using the BLASTP algorithm. The results are presented in Table 2. 

We were able to sequence seven from nine components which were short polypeptide chains from 7 to 14 amino acid residues in length. All identified peptides were linear, cysteine-free, and mainly enriched with Asp/Glu as well as polar and non-polar uncharged residues. This was due to the amino acid composition results. Searching for possible homologous sequences among Viridiplantae (taxid: 33,090) in whole, and *Equisetum* spp. (taxid: 3257) in particular, identified three peptide fragments of ribulose-1,5-bisphosphate carboxylase, a key plant enzyme located in chloroplasts. 

There were two short peptide fragments that were homologous to aquaporins and chitinases. These are proteins with defense functions. They are mainly inducible and responsible for external biotic stress, in particular fungal infections by pest damage that have not yet been studied. There were no peptides that could be gene-encoded, only fragments of protein hydrolysis. Finally, the molecular masses were measured for the dominant individual peptides located in a range from 600 to 1500 Da, whereas the total peptide extract was composed of 500–900 Da on average. This phenomenon can be associated with the ionization peculiarities of the peptides in a mixture compared to the individuals; this was not excluded in the partial fragmentation.

## 3. Discussion

Here, we studied plant peptide extracts for activity against potato late blight. We used partial acidic hydrolysis to obtain short peptides with relatively predetermined molecular masses. These peptide extracts were previously shown to have cytotoxic and antitumor activities in vitro and in vivo models [35,36]. The main functional aspect of this study consisted of screening these extracts for inhibition activity toward oomycete *P. infestans*—the causative agent of potato and tomato late blight—which is one of the most economically important crop diseases leading to significant losses of plant production annually around the world [37]. 

Considering this, natural target extracts obtained from accessible plant species are potentially good additions to chemical fungicides to decrease the residual quantities inside the final production of plant cultivation and the environment as a whole, and to make the technology of integrated plant protection less expensive. Our findings show that peptide extracts against *P. infestans,* as determined by optical microscopy, had a high level of inhibition. There are some mentions confirming the application of aquatic and organic plant extracts to suppress *P. infestans* and other phytopathogenic oomycetes; these are potent agents for biocontrol [38,39,40,41]. Generally, the target activity is associated with secondary metabolites of a different chemical nature and not with polypeptides.

It is typical to provide testing of the anti-oomycete activity of the peptide extracts to suppress the growth and development of *P. infestans,* including infection of the potato tuber surface. We could not confirm the same results; only a limited number of extracts displayed a strong level of pathogen deterrence in a time-dependent manner. These results are not surprising because the addition of plant tissue (slices of potato tubers) to the experiment led to the appearance of molecular interactions between the plant, its specific pathogen, and the external agent (a peptide extract). The activity was less effective because *P. infestans* could increase in the virulence level.

Several studies reported the molecular mechanisms of the Solanaceae plant’s resistance to *P. infestans* based on genomic and transcriptomic data analysis [42,43], the expression of genes encoding matrix metalloproteinases, the miRNA [44,45,46], and the inducible gene expression coding PR-proteins [47,48,49]. Taking another view, *P. infestans* is known to infect plant hosts that can realize protein virulence factors and that are activated in contact with a plant surface. These factors are enzymes (cyclophilins, phospholipases, and cutinases) [50,51,52], certain extracellular proteins that are represented by serine- and metalloproteinases [53], and a widely represented class of infection-associated effector proteins (with an *N*-terminal RXLR motif) [54,55].

We note that all antimicrobial peptide extracts were obtained via the efficient method of peptide material extraction. This provided a nearly complete absence of certain admixtures, including carbohydrates, bioflavonoids, steroids, glycosides, vitamins, and other theoretical dissoluble natural secondary metabolites [36]. Thus, all peptide extracts obtained using this original method were reproduced on their composition. These findings might have commercial value in the case of potential applications in industry.

The peptide extract isolated from common horsetail (*E. arvense*) demonstrated the most pronounced suppression of *P. infestans* compared to the other plants via two independent assays. This plant is primitive and ancient and belongs to the Equisetaceae family from the Polypodiophyta division. It is a good source of natural compounds, including antioxidants [56], flavonoids [57], and biologically-active carbohydrates [58]. *E. arvense* is a model object to isolate for the structural analysis of key proteins involved in the plant metabolism, including ferredoxin I and II isoforms [59], and cytochrome *c* reductase [60]. There currently is not any peptide isolated from common horsetail reported, including antimicrobials.

Previous authors reported that ethanolic extracts from *E. arvense* induced anticancer effects to suppress lung carcinoma cells at 100–150 µg/mL [61]. Hydroethanolic extracts were more effective as antinociceptive and anti-inflammatory agents in mice [62] and had a negative influence on *Aspergillus flavus* and *Fusarium verticillioides*; these produce mycotoxins in stored maize kernels (*Zea mays*) [63]. Aquatic extracts of *E. arvense* could possess antibacterial action toward clinical *Escherichia coli* involving the disruption of the biofilm formation [64].

Our results are the first mention that antimicrobial substances derived from *E. arvense* are peptides. A comparison of reversed-phase and anion-exchange HPLC for the isolation of compounds from the *E. arvense* peptide extract suggested that they are relatively hydrophilic and enriched by negatively charged amino acid residues. The active peptide extract contained a set of major components—the majority of which were fragments of partial hydrolysis of chloroplast proteins. Inside the extract, we identified peptide fragments that were members of the three main groups of plant proteins: aquaporins [65], chitinase A [66], and ribulose-1,5-bisphosphate carboxylase/oxygenase (Rubisco) [67].

Plant chitinases are known to have defense functions and are responsible for biotic stresses; however, the enzyme from common horsetail (*E. arvense*) belongs to class IIIb, for which no antifungal activity was detected. Thus, we may suppose that the antitumor activity previously detected [36], as well as the anti-oomycete effect described in this work may be associated with the target conversion of Rubisco to produce a number of biologically active anionic peptides with possible regulatory functions.

## 4. Materials and Methods

### 4.1. Medicinal Plants

To screen for antifungal activity in vitro, we selected plants used in traditional medicine: greater celandine (*Chelidonium majus*), PE-Cm; elecampane (*Inula helertium*), PE-Ih; common horsetail (*Equisetum arvense*), PE-Eqi; sweet bay (*Laurus nobilis*), PE-Ln; green tea (*Camellia sinensis*), PE-Cs; touch-and-heal (*Hypericum perforatum*), PE-Hp; and a mixture of the three plants combined (elecampane, greater celandine, and common horsetail), PE-Pm. Initially, all plant raw material was collected manually [35,68].

### 4.2. Oomycete Origin and Cultivation Conditions

The oomycete *P. infestans* strain OSV 12 was received from the Institute of Plant Protection (Priluki, Minsk District, the Republic of Belarus). Colonies of *P. infestans* were being grown in oat nutrient growth medium at the temperature of 14–16 °C for 12–14 days.

### 4.3. Peptide Extracts

To obtain the peptide extracts, the original procedure was performed to remove all high-molecular weight components (preliminary proteins and carbohydrates) and secondary metabolites. Taking 10 g of the raw plant, over ground material, was ground, soaked in 500 mL of 1 M acetic acid (Merck, Darmstadt, Germany), and left for 1 h at room temperature. During the exposition, the suspension was obtained using ultrasonication (five times through 10 min). Subsequently, the suspension was heated at 100 °C for 30 min, cooled, and centrifuged (75,000× *g*, 40 min, using a J2-21 (Beckman, Krefeld, Germany). Acetone was added to collect the supernatant at a ratio of 2:5 and precipitated at +4 °C overnight. Then, a pellet was separated by centrifugation (40,000× *g*, 40 min, J2-21 (Beckman, Germany). To improve the dissolving of a peptide extract, the pellet was dissolved in 100 mM acetic acid and repeatedly centrifuged (twice, 40,000× *g*, 20 min). To remove residual acidic quantities, the pellet was lyophilized twice by dissolving in 50 mL of MQ water.

### 4.4. Amino Acid Analysis

The amino acid composition was determined as described previously [36]. The analysis was performed on a Biotronik LC-3000 amino acid analyzer (Biotronik, Berlin, Germany). Regarding each reaction, 300 µg of the extract was selected for further acidic hydrolysis. Ion exchange HPLC was performed on a LC 3000 amino acid analyzer (Biotronik, Berlin, Germany). The peptide extracts were dissolved in 60 μL of a buffer containing 0.82 g of sodium acetate, 75 mL of methanol, 0.5 mL of glacial acetic acid, 100 μL of caprylic acid, and 10% formic acid in 1 L (solvent A), where the concentration of the peptide extracts was 10 mg/mL, and applied in a 1.0 × 125 mm column with VT-2410 resin (Biotronik, Berlin, Germany) equilibrated with solvent A.

Concerning elution, we used: solvent A, containing 1 g of 8.2 g of sodium acetate, 75.0 mL of methanol, 5.0 mL of glacial acetic acid, 4.0 mL of formic acid, and 100 μL of caprylic acid, pH 3.3; buffer containing 1 L 4.0 g of sodium hydroxide, 2.7 mL of glacial acetic acid, 2.5 mL of formic acid, and 100 μL of caprylic acid, pH 3.6 (solvent B); buffer containing 4.0 g of sodium hydroxide in 1 L, 2.7 mL of glacial acetic acid, 2.5 mL of formic acid, and 100 μL of caprylic acid, pH 4.9 (solvent C); and buffer containing 1 L of 1.0 mL of glacial acetic acid, 83 mM trisodium phosphate, 1.0 g EDTA, and 100 μL caprylic acid, pH 10.5 (solvent D). The samples were eluted sequentially with solvents A (30.5 min at 40 °C), B (7.5 min at 60 °C), C (12 min at 60 °C), and D (27 min at 60 °C) at a flow rate of 0.2 mL/min. To visualize, a ninhydrin solution containing 20 g of ninhydrin, 0.6 g of hydrindantin din, 50 mL of phenol, 550 mL of ethylene glycol monomethyl ether, and 400 mL of 4 M sodium acetate (pH 5.5) was used. The detection was monitored at 570 nm.

### 4.5. MALDI TOF Mass Spectrometry

The mass spectra were measured using an Ultraflex TOF/TOF instrument (Bruker, Berlin, Germany) in a positive ion mode. The peptide extracts were dissolved in 30% MeCN at a final concentration of about 500 pmoles/µL, and 1 µL was mixed with an equal volume of a matrix (2,5-dihydroxybensoic acid) (Merck, Kenilworth, NJ, USA), dried in air prior to analysis. The data obtained were processed with FlexAnalysis software.

### 4.6. Ion-Exchange HPLC

Ion-exchange separation was provided using the Agilent 1200 Series (Agilent Technologies, Santa Clara, CA, USA). The peptide extracts (0.5 mg) were dissolved in 50 µL of 50 mM PBS buffer (pH 8.0) and applied on a Luna SAX 4 × 250 mm column (Phenomenex, Torrance, CA, USA) equilibrated with the same solvent (buffer A). Peptides were eluted from the column using a linear gradient of buffer B (PBS and 500 mM NaCl, pH 8.0) at a flow rate of 1.0 mL/min. UV detection was monitored at 220 nm.

### 4.7. Reversed-Phase HPLC

To perform RP-HPLC, a Du Pont 8800 instrument (Du Pont, Wilmington, DE, USA) was used. The peptide extracts (0.5 mg) were dissolved in 50 µL of 0.1% trifluoroacetic acid (TFA) and applied on a Nucleosil^®^ 5/C18 4 × 250 mm column (Macherey–Nagel GmbH and Co, Düren, Germany) equilibrated with 0.1% TFA (buffer A). The peptides were eluted from the column using a linear gradient of buffer B (80% MeCN, 0.1% TFA) at a flow rate of 1.0 mL/min. The UV detection was monitored at 220 nm (Uvicord SII detector, GE HealthCare, Madison, WI, USA).

### 4.8. Edman Sequencing

*N*-terminal amino acid sequencing was conducted on a PPSQ-33A sequencer (Shimadzu, Kyoto, Japan) according to the manufacturer’s protocol. Approximately 500 pmoles of each compound purified by HPLC was analyzed. The identification of amino acid residues was performed using PTH derivatives using LabSolutions software.

### 4.9. Antifungal Assays In Vitro

The antifungal activity of the peptide extracts was tested using a microtiter-plate assay by measuring the inhibition of the germination of zoosporangiums as described [69]. Two-fold dilutions of extracts in pure water were prepared (from 2000 to 125 μg·mL^−1^) and added to the zoosporangium suspension in the water, containing, on average, from 5000 cells/mL. The plates were incubated at 15 °C and scored after 4 h (output of zoospores/direct germination) and 48 h (zoosporangium germination/indirect germination). Inhibition of the zoospores output (indirect germination) was estimated as the number of remaining zoospores relative to the total number (Each replicate is represented by calculation of cell quantity on 10 visual fields at optical microcopy). The inhibition of zoosporangium germination (direct germination) was estimated as the number of germinated zoosporangiums relative to their total number. These rates were expressed in ICmin (effect of inhibition more than 15%) and IC_50_ values showing the plant extract concentration required for the growth inhibition of 50% of the remaining zoospores or zoosporangium. The morphological changes in the microorganisms also were recorded. The experiments were conducted using two biological repetitions in three replicates of each variant.

### 4.10. Antifungal Assay by Inoculation of Potato Tuber Discs

The biological activity of peptide extracts also was assayed by estimating the degree of inhibition of *P. infestans* development on potato tuber discs [70]. Two potato tuber discs of similar size were placed in each Petri dish. The peptide samples were mixed with 50 µL of zoosporangium suspension in distilled water (at an average of 5000 zoosporangiums mL^−1^) to a final peptide extract concentration of 0.125–2.0 mg/mL and incubated at 20 °C for 2 h. The plant extract sample was applied to the center of each potato tuber disc. Potato discs infected with the zoosporangium suspension without peptide extract served as controls. The Petri dishes with infected potato tuber discs were incubated at 20 °C for 120 h. The disease severity was assayed 96, 120, and 144 h after inoculation by measuring the infected area of each disc and scored, denoting the absence of inhibition compared with the controls. A degree of infection is determined by comparison of a relative square of damage according to a full square of a tuber disc based on Abode Photoshop for Windows software. Ten discs were analyzed in each of three independent experiments.

## Figures and Tables

**Table 1:** Biological assays of peptide extracts on direct and indirect germination of *Phytophthora infestans* with application of microtiter-plate assay.

Index/Variant		Extract						
		PE-PM	PE-Eqi	PE-Cm	PE-In	PE-Hp	PE-Ln	PE-Cs
Indirect germination	IC_50_, mg/mL	0.25	1.0	-	-	2.0	-	2.0
	IC_min_, mg/mL	0.125	0.5	-	-	1.0	-	1.0
Direct germination	IC_50_, mg/mL	0.5	2.0	-	1.0	0.5	-	0.5
	IC_min_, mg/mL	0.125	1.0	2.0	0.5	0.25	2.0	0.25
Morphological changes		+	-	-	-	-	-	-

^“+”^—morphological changes of zoosporangiums: partial destruction and covering lysis at level of 15–18% at concentration of 2.0 mg/mL.

**Table 2:** A list of peptides identified in the extract from common horsetail (*E. arvense*).

No Peak	Average Molecular Mass Measured, Da	*N*-terminal Amino Acid Sequence	Annotation
1	1048.6	^1^PAVTLAFATTG^11^ *	Aquaporin product
2	905.2	^1^PSGGALNY^8^	Unidentified protein product
3	1160.4	^1^PVAGEDNQFLA^11^	Ribulose-1,5-bisphosphate carboxylase/oxygenase large subunit fragment
4	615.2	No sequence determined	-
5	943.5	^1^DFYRDND^7^	Possible hypothetical chloroplast RF21 protein fragment
6	1378.2	^1^DLIRDDFIEKD^11^	Ribulose-1,5-bisphosphate carboxylase/oxygenase large subunit fragment
7	1471.8	^1^QYQLDGIDLDYE^12^	Chitinase A fragment
8	1010.1	^1^DFTRDDEN^8^	Possible ribulose-1,5-bisphosphate carboxylase/oxygenase large subunit fragment
9	722.8	No sequence determined	-

* A number of amino acid residues in *N*-terminal sequences is marked by superscript.

## Abbreviations

MALDI-TOF MS	matrix-assisted laser desorption/ionization time-of-flight mass spectrometry
Asn	asparagine
Asp	aspartate
Gln	glutamine
Glu	glutamate
HPLC	high performance liquid chromatography
IC50	inhibition concentration caused half-effect
ICmin	inhibition concentration caused sub-effect (15%);
NCBI	National Center of Biotechnology Information, USA
BLASTP	basic local alignment search tool protein;
MQ	deionized water
EDTA	ethylenediaminetetraacetic acid
PBS	phosphate-buffered saline
NaCl	sodium chloride
UV	ultraviolet
RP-HPLC	reversed-phase high performance liquid chromatography;
MeCN	acetonitrile
PTH	phenylthiohydantoin
